# Supplementary material for: Modelling bioactivities of combinations of whole extracts of edibles with a simplified theoretical framework reveals the statistical role of molecular diversity and system complexity in their mode of action and their nearly certain safety
Source: PLoS One. 2020 Sep 28;15(9):e0239841. doi: 10.1371/journal.pone.0239841 (PMC7521709; doi:10.1371/journal.pone.0239841)
Supplement: Attachment — Submitted filename: Response to Reviewers.pdf [file pone.0239841.s001.pdf]

Pascal Mayer, PhD
Alphanosos
20-22 rue Henri et Gilberte Goudier, 63200 Riom, France
+33 659 856 881
Pascal.mayer@alphanosos.com

Zsoka Murakozi
Jed N. Lampe, Ph.D. Academic Editor
Dr. Joerg Heber, Editor-in-Chief
PLOS ONE

PONE-D-20-03593

Riom, July 23rd, 2020

Dear Dr Murakozi, Dr Lampe, dear Dr Heber

I am pleased to provide you with the revised manuscript of the original research article entitled "Modelling bioactivities of combinations of whole extracts of edibles with a simplified theoretical framework reveals the statistical role of molecular diversity and system complexity in their mode of action and their nearly certain safety" by Pascal MAYER for consideration for publication in PLOS ONE.

All editorial requirements have been addressed, namely:

1. Please ensure that your manuscript meets PLOS ONE's style requirements, including those for file naming.

The title-authors page has been reformatted according to
https://journals.plos.org/plosone/s/file?id=ba62/PLOSOne_formatting_sample_title_authors_affiliations.pdf
the main body, including format of bibliography, according to
https://journals.plos.org/plosone/s/file?id=wjVg/PLOSOne_formatting_sample_main_body.pdf and

2. We noted that one of your references did not auto populate and instead the manuscript contains the following "Error! Reference source not found", please replace this with the appropriate reference during your next revision.

Corrections have been realized by replacing the automatic equation numbering by the corresponding actual numbers.

3. Thank you for stating the following financial disclosure:

This work was financially supported by Alphanosos.

The funders through the author being a funders' employee had a role in study design, data collection and analysis, decision to publish, and preparation of the manuscript.

The following patents, marketed products, or products in development are related to this work:
- Patent family, applied for in 71 countries, including WO2018083115 and US2019255136
- Ediceutical based antibiotics and anti-cancer actives (preclinical research)
- Cosmetic ingredients: Synherbs®4.5, Synherbs®5.1, Synherbs®5.2
- Veterinary hygiene products Effiskin®, PhytoPyo,®, CaniPerfect®
- Cosmetic product Mokaskin®
- Alternatives to antibiotics for agriculture (preregistration research)

Additional Editor Comments (if provided): Please make update the references in the Discussion and Introduction sections as indicated by reviewer 1 before final submission of manuscript.

References have been amended as requested (see point 5)

4. Thank you for stating the following in the Competing Interests section:

I have read the journal's policy and the authors of this manuscript have the following competing interests: shareholder and employee of Alphanosos

This does not alter our adherence to PLOS ONE policies on sharing data and materials.

5 Please make update the references in the Discussion and Introduction sections as indicated by reviewer 1 before final submission of manuscript.

Reviewer #1: This research 'Modelling bioactivities of combinations of whole extracts of edibles with a simplified theoretical framework reveals the statistical role of molecular diversity and system complexity in their mode of action and their nearly certain safety' is informative and practical. In my opinion, this manuscript could be accepted in this form, however, I recommend that authors update the references with new publications (In discussion and introductions parts).

I added relevant references to more recent publications. Now 60% of citations are from 2016-2020 instead of 40% previously (2020: 5 (12%) vs 1 (2%), 2019: 9 (21%) vs 7 (16%), 2018: 8 (19%) vs 6 (14%), 2017: 2 (5%), 2016 2 (5%) vs 1 (2%)). I kept the existing references as I felt important to stress that some of the issues they illustrate and/or justify are known and published since a long time

I would like to thank you and the reviewer #1 for your review and very relevant und useful comments which allowed for an improved manuscript. Thank you for your consideration and implication.

Sincerely,

Pascal Mayer, PhD
Alphanosos
